# Whole-genome sequencing and metagenomics reveal diversity and prevalence of *Listeria* spp. from soil in the Nantahala National Forest

**DOI:** 10.1128/spectrum.01712-24

**Published:** 2024-12-09

**Authors:** Jia Wang, Claire N. Schamp, Lauren K. Hudson, Harleen K. Chaggar, Daniel W. Bryan, Katie N. Garman, Mark Radosevich, Thomas G. Denes

**Affiliations:** 1 Department of Food Science, The University of Tennessee, Knoxville, Tennessee, USA; 2 Tennessee Department of Health, Nashville, Tennessee, USA; 3 Department of Biosystems Engineering and Soil Science, The University of Tennessee, Knoxville, Tennessee, USA; Health Canada, Ottawa, Canada

**Keywords:** *Listeria*, Nantahala National Forest, whole-genome sequencing, average nucleotide identity, metagenomics, soil factors

## Abstract

*Listeria* spp. are widely distributed environmental bacteria associated with human foodborne illness. The ability to detect and characterize *Listeria* strains in the natural environment will contribute to improved understanding of transmission routes of contamination. The current standard for surveillance and outbreak source attribution is whole-genome sequencing (WGS) of *Listeria monocytogenes* clinical isolates. Recently, metagenomic sequencing has also been explored as a tool for the detection of *Listeria* spp. in environmental samples. This study evaluated soil samples from four locations across altitudes ranging from 1,500 to 4,500 ft in the Nantahala National Forest in North Carolina, USA. Forty-two *Listeria* isolates were cultured and sequenced, and 12 metagenomes of soil bacterial communities were generated. These isolates comprised 14 distinct strains from five species, including *Listeria cossartiae* subsp. *cayugensis* (*n* = 8; *n* represents the number of distinct strains), *L. monocytogenes* (*n* = 3), “*Listeria swaminathanii*” (Lsw) (*n* = 1), *Listeria marthii* (*n* = 1), and *Listeria booriae* (*n* = 1). Most strains (*n* = 13) were isolated from lower altitudes (1,500 or 2,500 ft), while the *L. swaminathanii* strain was isolated from both higher (4,500 ft) and lower (1,500 ft) altitudes. Metagenomic analysis of soil described a reduction in both bacterial community diversity and relative abundance of *Listeria* spp. as the altitude increased. Soil pH and cation exchange capacity were positively correlated (*P* < 0.05) with the abundance of *Listeria* spp. as detected by metagenomics. By integrating culture-independent metagenomics with culture-based WGS, this study advances current knowledge regarding distribution of *Listeria* spp. in the natural environment and suggests the potential for future use of culture-independent methods in tracking the transmission of foodborne pathogens.

**IMPORTANCE:**

As a foodborne pathogen, *Listeria* continues to cause numerous illnesses in humans and animals. Studying the diversity and distribution of *Listeria* in soil is crucial for understanding potential sources of contamination and developing effective strategies to prevent foodborne outbreaks of listeriosis. Additionally, examining the ecological niches and survival mechanisms of *Listeria* in natural habitats provides insights into its persistence and adaptability, informing risk assessments and public health interventions. This research contributes to a broader understanding of microbial ecology and the factors influencing foodborne pathogen emergence, ultimately enhancing food safety and protecting public health. Moreover, using a metagenomic approach provides a detailed understanding of the soil microbial ecosystems, leading to more effective monitoring and control of foodborne pathogens. This study also highlights the potential for integrating metagenomics into routine surveillance systems for food safety in the near future.

## INTRODUCTION

*Listeria* spp. are ubiquitous in natural environments such as soil and water, which can easily contaminate food processing environments. Listeriosis, the foodborne illness caused by *Listeria monocytogenes* (Lmo) is a global burden with substantial mortality (20%–30%) and a 94% hospitalization rate in the United States (1, 2). With the potential to cause nervous system failure in immunocompromised individuals and abortions in pregnant women, listeriosis outbreaks are considered difficult to control (3, 4) and represent a significant threat to public health (5, 6).

Many previous studies have demonstrated that pristine natural forests and mountains are reservoirs for *Listeria* spp. (7–10). Previous research has described the biodiversity of *Listeria* in national forests and national parks in the southern Appalachian region of the United States, where this study was conducted (11–14). It has been reported that soil moisture, molybdenum, and salt concentrations are related to *Listeria* incidence in natural soil ecosystems (15). *Listeria* in soil can contaminate farmland and irrigation water through rainfall-induced soil splash, potentially transferring to food products (16). Wildlife activities may also facilitate the spread of these pathogenic bacteria into areas of human activity (17). The strong adaptability of *Listeria* to diverse environmental conditions further contributes to its widespread distribution and varied transmission routes (18). The effort to monitor *Listeria* contamination events includes a more expansive look at both raw materials and natural environments in the farm-to-fork continuum and includes the intersection of natural and built environments (7, 8, 11, 19–21).

Understanding the ecology of pathogenic microorganisms can provide knowledge on how to develop mitigation strategies (22, 23). The isolation and genomic characterization of *Listeria* spp. from specific geographical locations will contribute to our understanding of the phylogenomics of *Listeria* spp. and serve as a source of biogeographical indicator strains that can populate source attribution databases (11, 24). Whole-genome sequencing (WGS) and subsequent high-resolution phylogenetic evaluation of complete genomes have provided a powerful method for the surveillance of foodborne pathogens (8). WGS can also provide highly discriminative genotypic information, which is helpful in evaluating the risk of *Listeria* transmission from the natural environment into agricultural and food processing environments (25, 26).

Ecological signatures of microbial communities provided by metagenomic data can further our understanding of how foodborne pathogens relate with their natural reservoirs and can aid in the identification of potential points of contamination (27, 28). Metagenomic sequencing is a culture-independent surveillance tool that can be used to identify pathogen-specific signatures (such as specific DNA sequences, functional genes, and antibiotic resistance genes) in microbial communities (29, 30). This approach characterizes microbiota of environmental samples and holds great potential for advancing epidemiological investigations of foodborne outbreaks (23). Integrated analysis of metagenomic and WGS data can also contribute to more accurate estimates and models of incidence and diversity of *Listeria* spp. and thus support the development of more advanced pathogen risk assessment and management approaches based on correlated genetic markers (31–34).

In the present study, a combination of WGS and culture-independent metagenomic methods was used to investigate the diversity of *Listeria* spp. in soil bacterial communities across four different altitudes in the Nantahala National Forest (NNF) in North Carolina, USA. Additionally, soil properties were evaluated to determine their correlation with soil microbial biodiversity and *Listeria* distribution. Our expectation was that *Listeria* spp. isolated from NNF would contribute to an improved understanding of the diversity of *Listeria* genotypes and potentially provide signatures for metagenomic analysis of uncommon *Listeria* spp. through alignment with a custom database of *Listeria* type and reference strains. This study provides insights into the persistence of *Listeria* spp. in the natural environment, and its findings may lead to an integrated surveillance regime to improve environmental monitoring of pathogenic strains of *Listeria* and bring early awareness of outbreaks that could occur.

## MATERIALS AND METHODS

### Sampling site and soil chemical properties

After a permit was issued by the United States Department of Agriculture (USDA) Forest Service, soil samples were collected on a single day, 27 September 2022, in the NNF near Robbinsville, NC, along the Appalachian Trail from the Nantahala River up to Cheoah Bald. Nantahala National Forest, the largest national forest in North Carolina, covers over 531,000 acres, with elevations ranging from 1,200 to 5,800 ft (366–1,768 m) (35).

**Fig 1:**
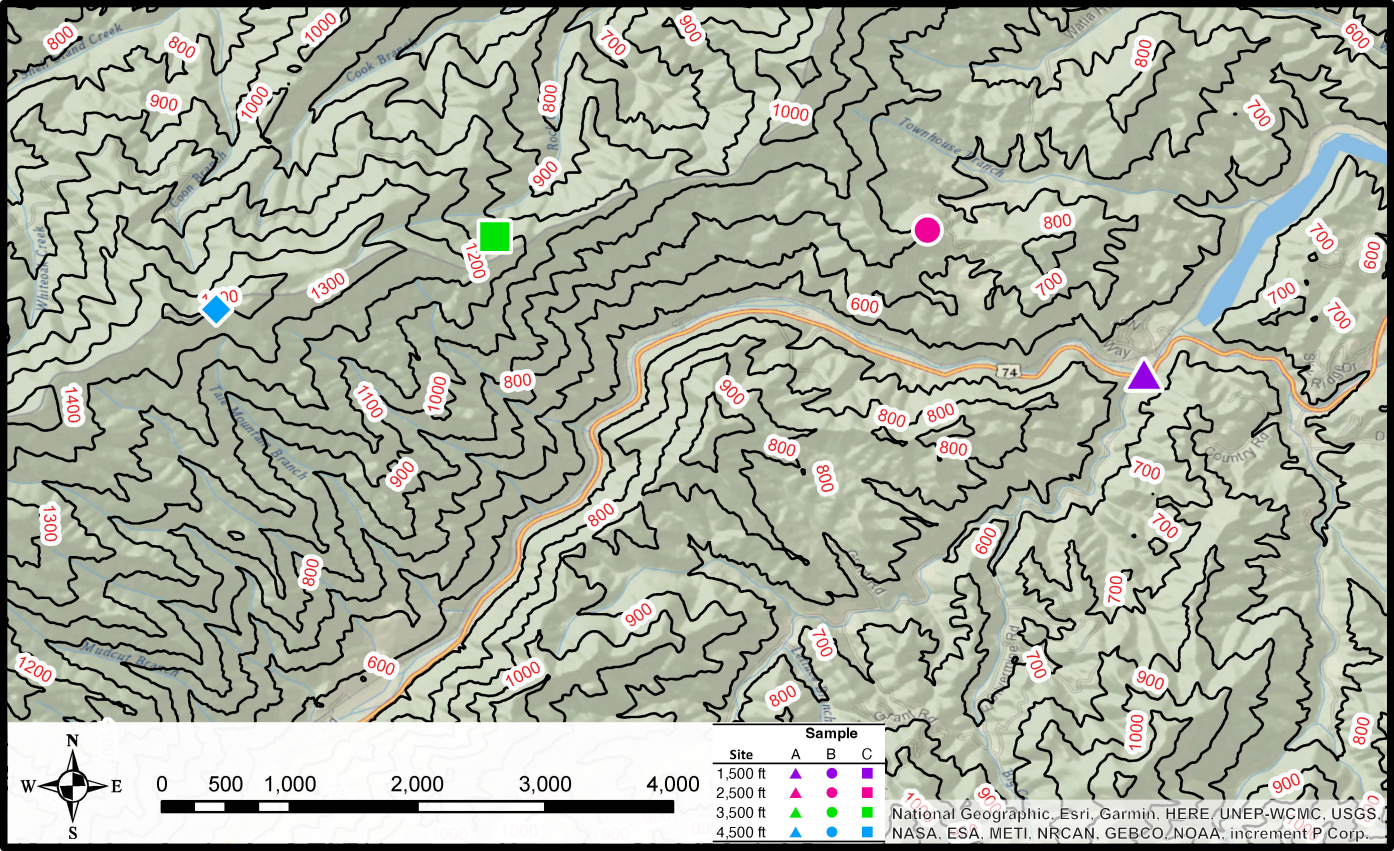
Map of soil sampling sites at four different altitudes in the Nantahala National Forest. This figure was created with ArcGIS Pro software (v.3.3; ESRI, Redlands, CA, USA) using topographical data from The National Map (https://topobuilder.nationalmap.gov/). Approximate site elevations are indicated by colored shapes: 1,500 ft (purple triangle), 2,500 ft (pink circle), 3,500 ft (green square), and 4,500 ft (blue diamond).

Four sampling sites were chosen based on 1,000-ft (~300 m) increments of elevation change (Fig. S1; Fig. 1). Three samples were collected at each sampling site/altitude, yielding 12 total. Each sample contained 500 g of soil from 1 m^2^ of area. Samples were homogenized, placed in sterile Whirl-Pak bags, and stored on ice throughout the hike and while in transit to the laboratory. Samples were stored overnight at 4°C and then sent to Midwest Laboratories (Omaha, NE, USA) for soil analysis using their S1AN package. Moisture content dry basis was determined for each soil sample and was determined gravimetrically by 5 g of soil and drying in an oven for 24 h at 107°C.

### *Listeria* enrichment and isolation

Soil samples were enriched for *L. monocytogenes* and other *Listeria* spp. (Fig. S1) using a standard method as previously described (15, 36). Modified Oxford Agar (MOX) (Thermo Fisher Scientific, Waltham, MA, USA) and Brilliance *Listeria* Agar Base (*Listeria monocytogenes* plating medium [LMPM]) plates (Oxoid Ltd, Basingstoke, Hampshire, United Kingdom) were used to isolate *Listeria* spp. and *L. monocytogenes,* respectively. Soil samples were stored for 60 h at 4°C prior to beginning the enrichment process.

Twenty-five grams of each soil sample was placed in sterile Whirl-Pak bags. Then 225 mL of buffered *Listeria* enrichment broth (BLEB) base (Difco, Sparks, MD, USA) was added to each sample. Samples were stomached for 60 s at 260 rpm and incubated for 4 h at 30°C in a stationary incubator. After 4 h, 900 µL of *Listeria* selective enrichment supplement (Oxoid Ltd) was added to the samples and incubated at 30°C for an additional 18 h. After 24 h, 50-µL aliquots were obtained and streaked for isolation on MOX and LMPM plates. MOX plates were incubated at 30°C, and LMPM plates were incubated at 35°C for 48 h. Enriched soil samples were incubated for an additional 24 and at 48 h, and this process was repeated. Up to four colonies showing characteristics of *Listeria* spp. and *L. monocytogenes* were isolated from each MOX and LMPM plate and re-streaked onto a new LMPM or MOX plate. This process was completed at both 24 and 48 h.

### Whole-genome sequencing and analysis for isolates

For each pure isolate (*n* = 72), genomic DNA was extracted with the DNeasy Blood & Tissue Kit (QIAGEN, Germantown, MD, USA) as previously described (37). The genomic DNA was shipped to SeqCenter (Pittsburgh, PA, USA) for sequencing. Sample libraries were prepared using the Illumina DNA Prep kit and IDT 10-bp UDI indices (Illumina, San Diego, CA, USA), and sequenced on an Illumina NextSeq 2000, producing 151-bp paired-end reads. Demultiplexing, quality control, and adapter trimming were performed with BCL Convert (v.3.9.3) (38).

Trimmomatic (v.0.36, with the following parameters: Leading 3, Trailing 3, SlidingWindow 4:15, and MinLen 36) was used to trim reads (39). Read quality was checked with FastQC (v.0.11.9) (40). Reads were assembled using SPAdes (v.3.13.1), and the resulting contigs were filtered to remove those that were <1 kbp in length or had <5× read coverage (41). QUAST (v.5.0.2) was used to calculate assembly statistics (42).

To determine the taxonomic classification of the isolates, PYANI (v.0.2.10, using BLAST+) (43) was used to calculate average nucleotide identity (ANI) values between the isolate assemblies and type, and representative *Listeria* strains were downloaded from the National Center for Biotechnology Information (NCBI) RefSeq or GenBank (Table S1). The resulting pairwise ANI values were then used as input to bactaxR to create a dendrogram (44). Type Strain Genome Server and GTDB-Tk on KBase were also used to check taxonomic placement. Groups of isolates with high ANI were each individually further evaluated using the CFSAN SNP Pipeline (v.2.2.1) to determine high-quality single-nucleotide polymorphism (hqSNP) distances between isolates (45). Isolates that were 0 hqSNPs apart were considered identical (i.e., indistinguishable, the same strain). Conversely, those with ANI values of <99.99% and ≥1 hqSNPs apart were considered distinct strains. For prevalence/abundance calculations, identical isolates that were isolated from multiple samples were considered once for each separate sample, indicating that the same strain was isolated from two different samples or sites. Additionally, the *L. monocytogenes* isolates were queried on the NCBI Pathogen Detection Isolates Browser to determine if there were any other closely related isolates in the database.

### Metagenomic sequencing and analysis

The total DNA in soil was extracted using the DNeasy PowerSoil Pro Kit (QIAGEN) from 250-mg soil of each sample according to the manufacturer’s instructions with slight modifications. The homogenization step was performed using a FastPrep-24 Classic bead-beating grinder and lysis system (MP Biomedicals, Irvine, CA, USA) with 2 × 30 s at 4 m/s. Due to difficulty isolating DNA from soil sample 2500A using DNeasy PowerSoil Pro Kit, DNA was extracted from 10-g soil using DNeasy PowerMax Soil Kit (QIAGEN) following the manufacturer’s protocols. The concentrations of DNA samples were determined using a NanoDrop One Spectrophotometer (Thermo Fisher Scientific Inc, Wilmington, DE, USA) and a Qubit four fluorometer with dsDNA BR Assay Kit (Thermo Fisher Scientific Inc).

The total DNA extracted from the soil samples was shipped to SeqCenter for sequencing. Sample libraries were prepared using the Illumina DNA Prep kit and IDT 10-bp UDI indices (Illumina) and sequenced on an Illumina NextSeq 2000, producing 151-bp paired-end reads. Demultiplexing, quality control, and adapter trimming were performed with BCL Convert (v.3.9.3) (38).

Paired-end metagenomic reads in FASTQ format were uploaded to Metagenomic Rapid Annotation using the Subsystem Technology (MG-RAST, v.4.0.3) online platform and processed to remove low-quality reads with default dereplication, screening, and trimming settings (46). Artificial duplicate reads were evaluated by duplicate read inferred sequencing error estimation. The taxonomy annotation of metagenomes was determined using MG-RAST by comparing to the NCBI RefSeq database (release date 10 November 2022) using the default parameters: maximum *e* value of 1e-5, a minimum ID value of 60%, and a minimum alignment length of 15 bp (47–52). For further analysis, the taxonomy table generated at different taxa levels was used, and the abundance of each taxon was represented in terms of percentage of total effective bacterial sequences in a sample. The metagenomic reads assigned as *Listeria* genus by MG-RAST were further aligned with a custom database using Geneious Prime (v.2023.0.4; Biomatters, Auckland, New Zealand) BLASTN (maximum *e* value set at 1e-5). This database included reference and type genomes of all *Listeria* spp. (*n* = 68), all representative *Listeria* genomes identified and sequenced by Liao et al. (15) (*n* = 594), and genomic assemblies of *Listeria* isolates from this study (*n* = 42) (Table S2).

### Statistical analysis

All statistical analyses in this study were performed using functions in R software (v.4.2.2) (http://www.r-project.org). The heatmap was created using pheatmap package, and the *Z*-score for the heatmap was calculated using the following formula: *Z* = (*x* – mean)/SD, where *x* is the reads of a class; mean is the mean value; and SD is the standard deviation. Pearson’s correlation and Euclidean distance were used for clustering analysis. Alpha diversity was calculated using vegan package from an operational taxonomic unit (OTU) table at genus level using Shannon and Simpson diversity indices. The Shannon index provides information on both the richness and evenness of a microbial community, making it crucial for understanding the overall diversity structure in soil samples. In contrast, the Simpson index gives more weight to dominant species. These diversity metrics are widely used in microbial ecology studies, facilitating comparisons with existing literature (53, 54). One-way analysis of variance (ANOVA) was used to analyze statistical significance of difference of alpha diversity indices. The pairwise correlations between soil physicochemical factors were determined via Pearson correlation analysis, and the significance level was adjusted for multiple comparisons by Bonferroni correction. The soil physicochemical factors analyzed included organic matter, total soil phosphorus, potassium, magnesium (Mg), calcium, pH value, cation exchange capacity (CEC), total organic carbon, and moisture content. A total of 45 comparisons were made between pairs of these factors. Detrended correspondence analysis (DCA) was used to evaluate the gradient size of the genus distribution in soil microbial communities to determine if redundancy analysis (RDA) would be appropriate. The length of the first DCA axis of the soil bacteria was less than 3; therefore, RDA could be a more suitable analytical method than canonical correspondence analysis (55, 56). RDA was conducted using vegan package to examine the relationships between bacterial communities and soil geochemical properties, and Euclidean dissimilarity was calculated using the reads of each genus detected by MG-RAST. The statistical significance of RDA was evaluated by 999 permutations. The variance inflation factor (VIF) of each soil variable was calculated, and the soil variable with the highest VIF value was removed. Then the VIF analysis was repeated until all the VIF values were ≤10 (57). Mantel analysis for correlation between soil properties and microbial composition was performed using “Mantel” function in vegan package and the significance analysis was based on 999 permutation tests. R package ggplot2 was used for generating graphs.

## RESULTS

### *Listeria* isolation, whole-genome sequencing, and phylogenetic analysis

**Fig 2:**
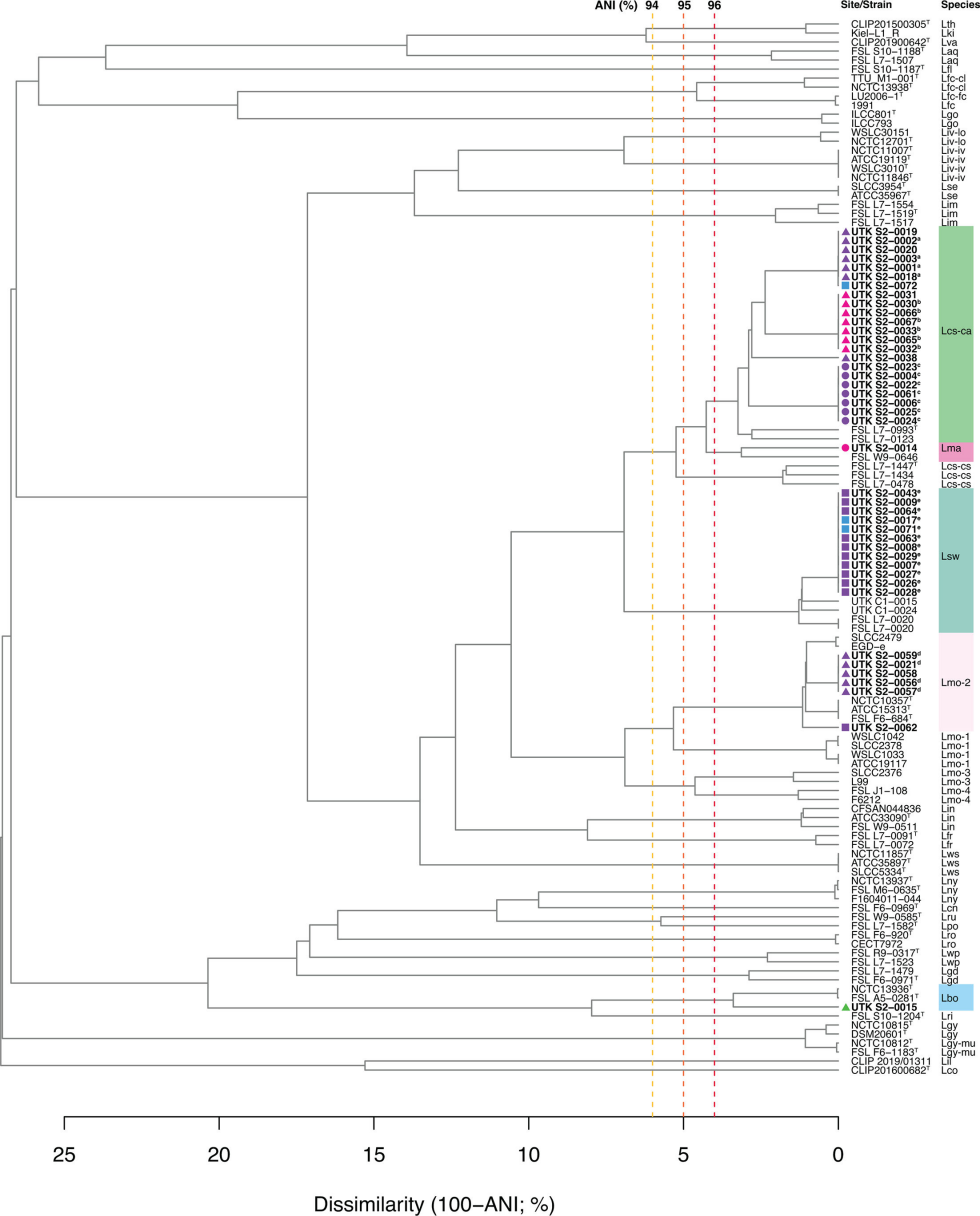
Average nucleotide identity (ANI) dendrogram of study isolates, type strains (strain name followed by a superscript letter T), and other reference strains. Sampling sites/elevations for strains isolated in this study are indicated by colored shapes defined in the legend at the bottom right of the figure. Dashed vertical lines indicate dissimilarities of 6% (yellow), 5% (orange), and 4% (red), which correspond to ANI values of 94%, 95%, and 96%, respectively. Species clusters that contain strains isolated in this study are indicated by colored boxes. Species designations are abbreviated as follows: *Listeria aquatica* (Laq), *Listeria booriae* (Lbo), *Listeria cornellensis* (Lcn), *Listeria cossartiae* subsp. *cayugensis* (Lcs-ca), *L. cossartiae* subsp. *cossartiae* (Lcs-cs), *Listeria costaricensis* (Lco), *Listeria farberi* (Lfr), *Listeria fleischmannii* subsp. *coloradonensis* (Lfc-cl), *L. fleischmannii* subsp. *fleischmannii* (Lfc-fc), *Listeria floridensis* (Lfl), *Listeria goaensis* (Lgo), *Listeria grandensis* (Lgd), *Listeria grayi* (Lgy), “*L. grayi* subsp. *murrayi*” (Lgy-mu), *Listeria ilorinensis* (Lil), *Listeria immobilis* (Lim), *Listeria innocua* (Lin), *Listeria ivanovii* subsp. *ivanovii* (Liv-iv), *L. ivanovii* subsp. *londoniensis* (Liv-lo), “*Listeria kieliensis*” (Lki), *Listeria marthii* (Lma), *Listeria monocytogenes* (Lmo), *Listeria murrayi* (Lmu), *Listeria newyorkensis* (Lny), *Listeria portnoyi* (Lpo), *Listeria riparia* (Lri), *Listeria rocourtiae* (Lro), *Listeria rustica* (Lru), *Listeria seeligeri* (Lse), “*Listeria swaminathanii*” (Lsw), *Listeria thailandensis* (Lth), *Listeria valentina* (Lva), *Listeria weihenstephanensis* (Lwp), and *Listeria welshimeri* (Lws). Isolates that are identical (>99.99% ANI and hqSNP distance of 0) share the same superscript lowercase letter following the isolate name.

Twelve soil samples were collected from four separate sites along an altitudinal transect in the NNF (Fig. 1). The soil samples yielded 42 isolates that were identified as *Listeria* spp. The other 30 isolates were determined to belong to other genera (including *Bacillus*, *Glutamicibacter*, *Lysinbacillus*, and *Niallia*) and will not be further discussed. The genome sizes for the 42 *Listeria* isolates sequenced in this study ranged from 2.73 to 3.97 Mbp in length with GC content from 37.82% to 41.57%. The completeness of 42 *Listeria* genomes was estimated as no less than 96.69%, and their contamination was no more than 1.10% (Table S3). The dendrogram, made by average nucleotide identity BLAST (ANIb) of the assemblies, showed that the 42 *Listeria* isolates consisted of five different *Listeria* species (Fig. 2; Table S4). All genomes had ANI values higher than 96% with their corresponding species type strain genomes, which were above the 95% similarity threshold for differentiating two species (58).

The 42 *Listeria* isolates consisted of five different species and 14 unique strains. Most (22 isolates) were identified as *Listeria cossartiae* subsp. *cayugensis* (Lcs-ca). These isolates consisted of eight distinct strains (those that were >99.99% ANI and with 0 hqSNPs apart were considered the same strain (11), and there were three groups of identical isolates (Fig. 2). One isolate, UTK S2-0014, from a 2,500-ft (762 m) soil sample, was identified as *Listeria marthii* (Lma). Twelve isolates were identified as Lsw, and among them, 10 isolates were from an elevation of 1,500 ft (457 m) and 2 were from 4,500 ft (1,372 m); these isolates were all identical, so they consisted of a single distinct strain. However, this single strain was isolated from two different soil samples, so this was considered as two separate isolation events. Six isolates from 1,500-ft sites were identified as *Listeria monocytogenes*, specifically belonging to lineage II; these isolates consisted of three distinct strains. These *L. monocytogenes* isolates were queried on the NCBI Pathogen Detection Isolates Browser to determine if there were any other closely related isolates in the database; five of them belonged to and were the only current database isolates in SNP cluster PDS000159743.1. Additionally, one isolate, UTK S2-0015, was identified as *Listeria booriae* (Lbo); it was isolated from a sample taken at 3,500 ft (1,067 m). In summary, of the 42 *Listeria* isolates, 14 distinct strains (ANI <99.99% and hqSNPs >0) were identified from 15 separate isolations (Table S5).

### Analysis of shotgun metagenomic sequencing data

All the metagenomes attained saturation in the rarefaction curves (Fig. S2), suggesting that the sequencing depth for all metagenomes was sufficient for good representative coverage of the microbial diversity in the soil samples. The metagenome data characteristics of the 12 soil samples (after quality control processing in MG-RAST) are detailed in Table S6.

### Composition and diversity of soil bacterial communities

**Fig 3:**
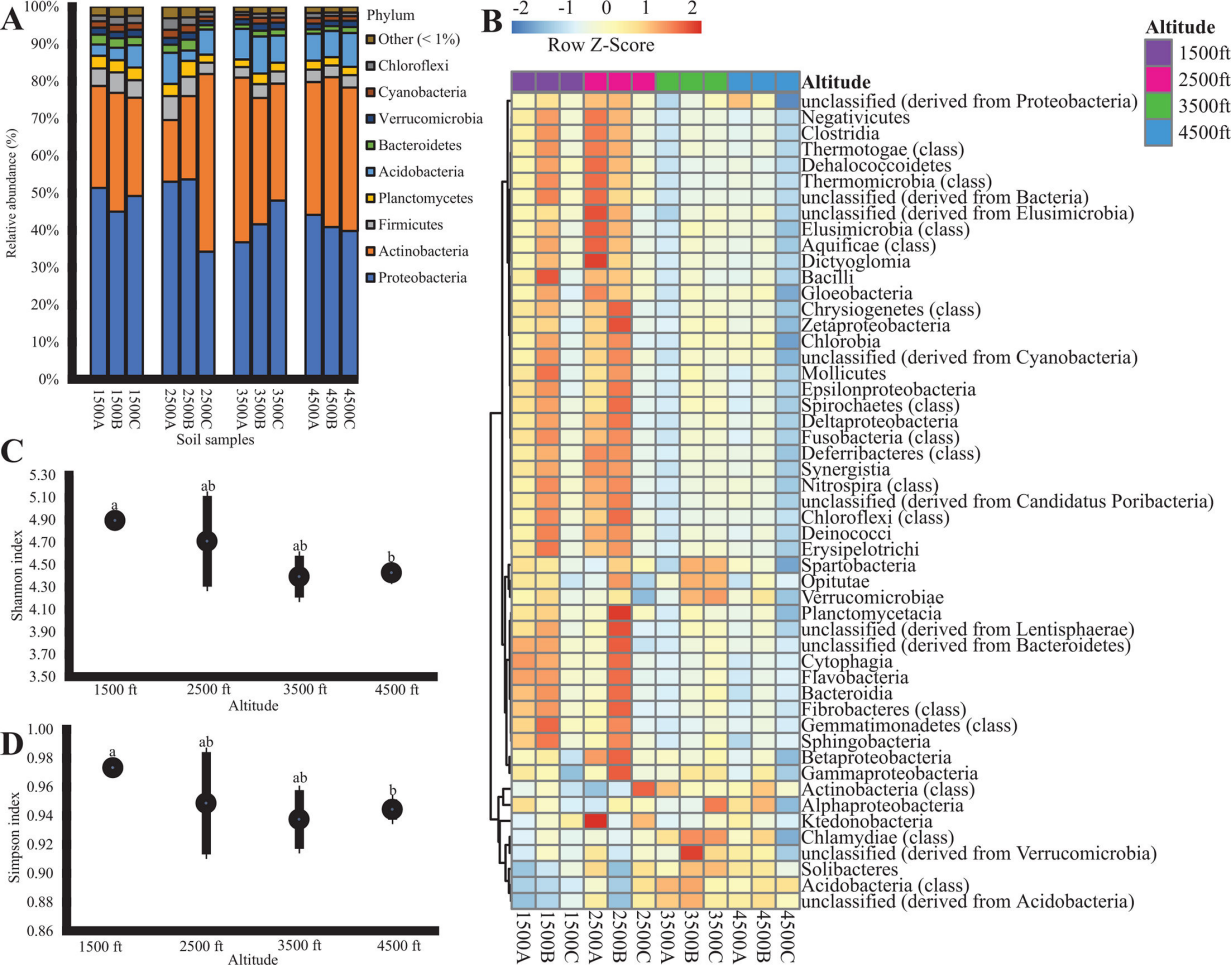
Bacterial community composition and diversity in soil samples. (**A**) Relative abundances of bacterial phyla in all soil samples from different altitude levels. The order of phyla listed in the figure legend corresponds to the sequence of stacked segments from top to bottom in each column of the chart. (**B**) Heatmap containing the abundances of different classes detected in the soil samples at different altitudes. Clustering analysis was performed using the Pearson correlation coefficient and Euclidean distance. Each row in the heatmap was standardized by *Z*-score, and the legend indicates the *Z*-score of the abundance of a class. (**C**) Shannon index and (**D**) Simpson diversity index of bacterial communities at genus level resolution. Each data point represents the mean, and error bars represent the standard error of triplicate samples. Different letters above each point indicate statistically significant differences (*P* < 0.05) in index among groups, and points with the same letter indicate that there is no statistical difference among them.

Phylum level analysis of the metagenomes showed that more than 30 phyla were detected in the soil samples taken from different altitude levels. Proteobacteria, Actinobacteria, Firmicutes, Planctomycetes, Acidobacteria, Bacteroidetes, Verrucomicrobia, Cyanobacteria, and Chloroflexi (mean relative percentage value >1%) represented the most prevalent phyla and were ubiquitously distributed at all altitudes. These phyla contributed more than 97% of the identified sequences (Fig. 3A). Proteobacteria and Actinobacteria were most abundant (>10%) in all soil samples. The relative abundance of Proteobacteria was higher in the bacterial community of soil from the lower altitude (1,500 ft; 48.52%) compared to that from the higher altitude (4,500 ft; 41.59%). Conversely, the relative abundance of Actinobacteria was higher at 4,500 ft (38.12%) than at 1,500 ft (28.55%) (*P* < 0.05). Firmicutes and Planctomycetes were significantly more abundant at the lowest elevation (1,500 ft; 4.95% and 3.34%) than they were at 3,500 ft (3.26% and 2.43%) and 4,500 ft (3.28% and 2.30%), whereas Acidobacteria was more abundant at higher altitudes (3,500 ft; 8.47% and 4,500 ft; 7.77%) than at lower altitudes(1,500 ft; 4.14%). At the class level, a total of 51 classes were identified throughout all soil metagenomes, and for most classes, higher abundance occurred at relatively lower altitudes (1,500 and 2,500 ft) (Fig. 3B). Spartobacteria, Verrucomicrobiae, Alphaproteobacteria, Chlamydiae, unclassified Verrucomicrobia, Solibacteres, Acidobacteria, and unclassified Acidobacteria increased in relative abundance at higher altitudes (3,500 or 4,500 ft), while the relative abundance of all other classes declined as altitude increased.

Shannon and Simpson indices were used to quantify bacterial diversity in soil samples from different altitudes. According to both indices, the bacterial community diversity was highest at the 1,500-ft level, while less diversity was observed at the 4,500-ft level (Fig. 3C and D). The ANOVA test showed significant difference (*P* < 0.05) in both Shannon and Simpson diversity mean values between 1,500 and 4,500 ft. The bacterial community diversity at 2,500 and 3,500 ft had no statistically significant difference (*P* > 0.05) from any of the other altitude levels.

### Distribution of *Listeria* spp. across different altitudes and comparison between culture-dependent and culture-independent approaches

**Fig 4:**
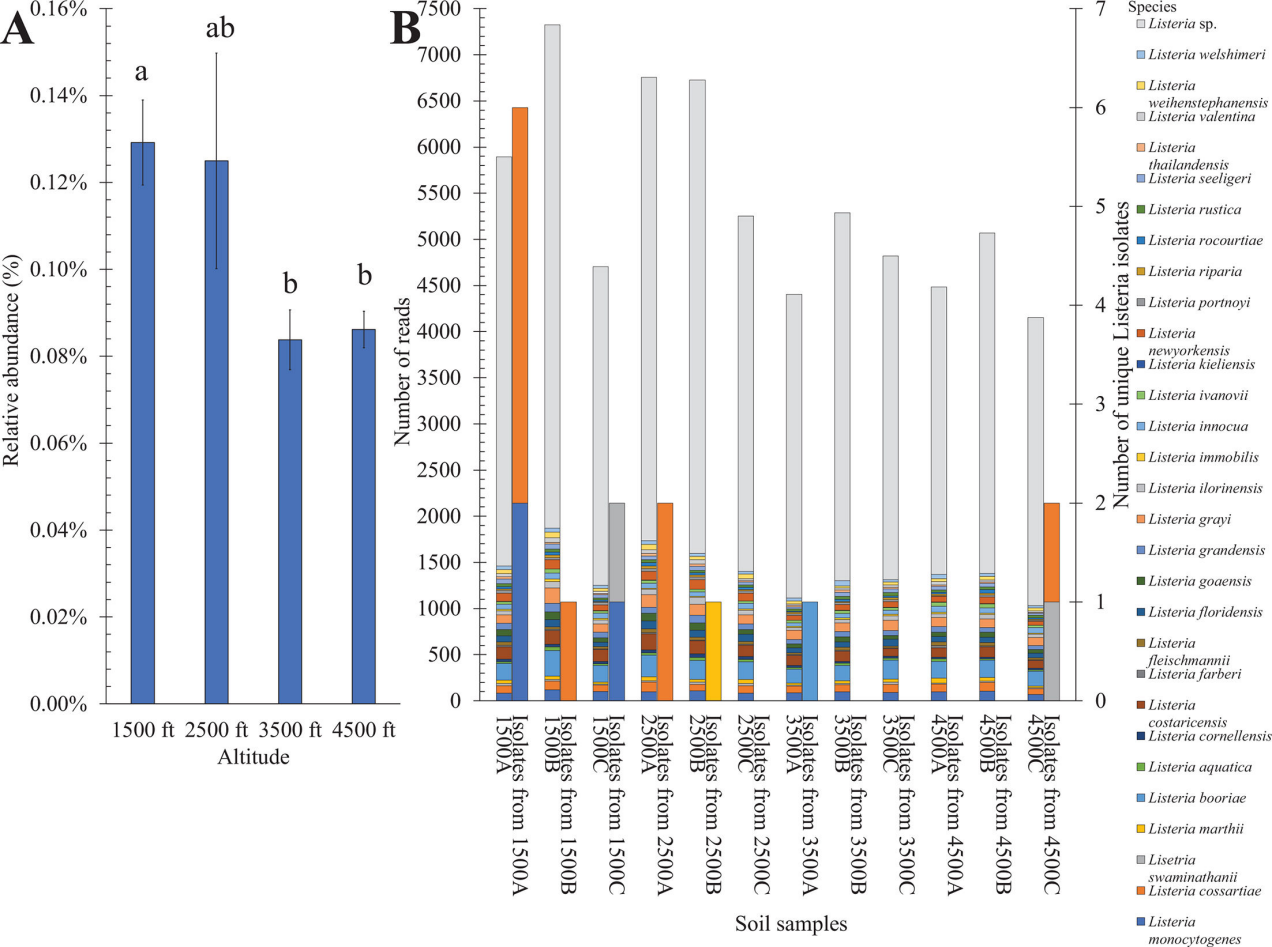
*Listeria* detected by metagenomic analysis in soil samples from different altitudes in the Nantahala National Forest. (**A**) *Listeria* relative percentages in soil samples. Each data column represents the mean, and error bars represent the standard error of triplicate samples. Different letters above each column indicate statistically significant differences (*P* < 0.05) in *Listeria* relative abundance among groups. (**B**) Relative percentages of *Listeria* strains at species level in each soil sample from BLASTN to a custom *Listeria* genome database, and the number of distinct *Listeria* strains isolated from soil samples.

The soil metagenomes were analyzed for the presence of *Listeria* spp. by the MG-RAST web tool. All metagenomes showed low abundance of *Listeria* genus (relative abundances <0.2%). The percentage of *Listeria* reads was the highest at lower altitudes (1,500 and 2,500 ft) and decreased significantly (*P* < 0.05) at higher altitudes (3,500 and 4,500 ft) (Fig. 4A; Table S7), in line with the notable decrease in overall diversity of bacterial communities found at 3,500- and 4,500-ft altitudes.

As determined by MG-RAST, the reads belonging to the *Listeria* genus were subsequently aligned to a custom *Listeria* genome database, which consisted of genomes of representative and type strains of all known *Listeria* spp., the *Listeria* isolated in this study (*n* = 42), and other representative isolates. This comprehensive analysis further strengthened the evidence supporting the presence of *Listeria* spp. in these metagenomes. The number of *Listeria* reads in the metagenomes (taxonomically classified as *Listeria* by BLAST) was compared with the number of distinct *Listeria* isolates at different altitudes, and both showed similar trends by altitude (Fig. 4B). Moreover, the metagenomic reads classified as *Listeria* at the species level included all five identified *Listeria* spp. in this study and could be aligned with genomes of nine of the distinct *Listeria* strains isolated in this study (Table S8). However, the reads were also identified as 24 other species that were not isolated from the soil samples (Fig. 4B).

### Effect of environmental characteristics on bacterial community and *Listeria* composition

**Fig 5:**
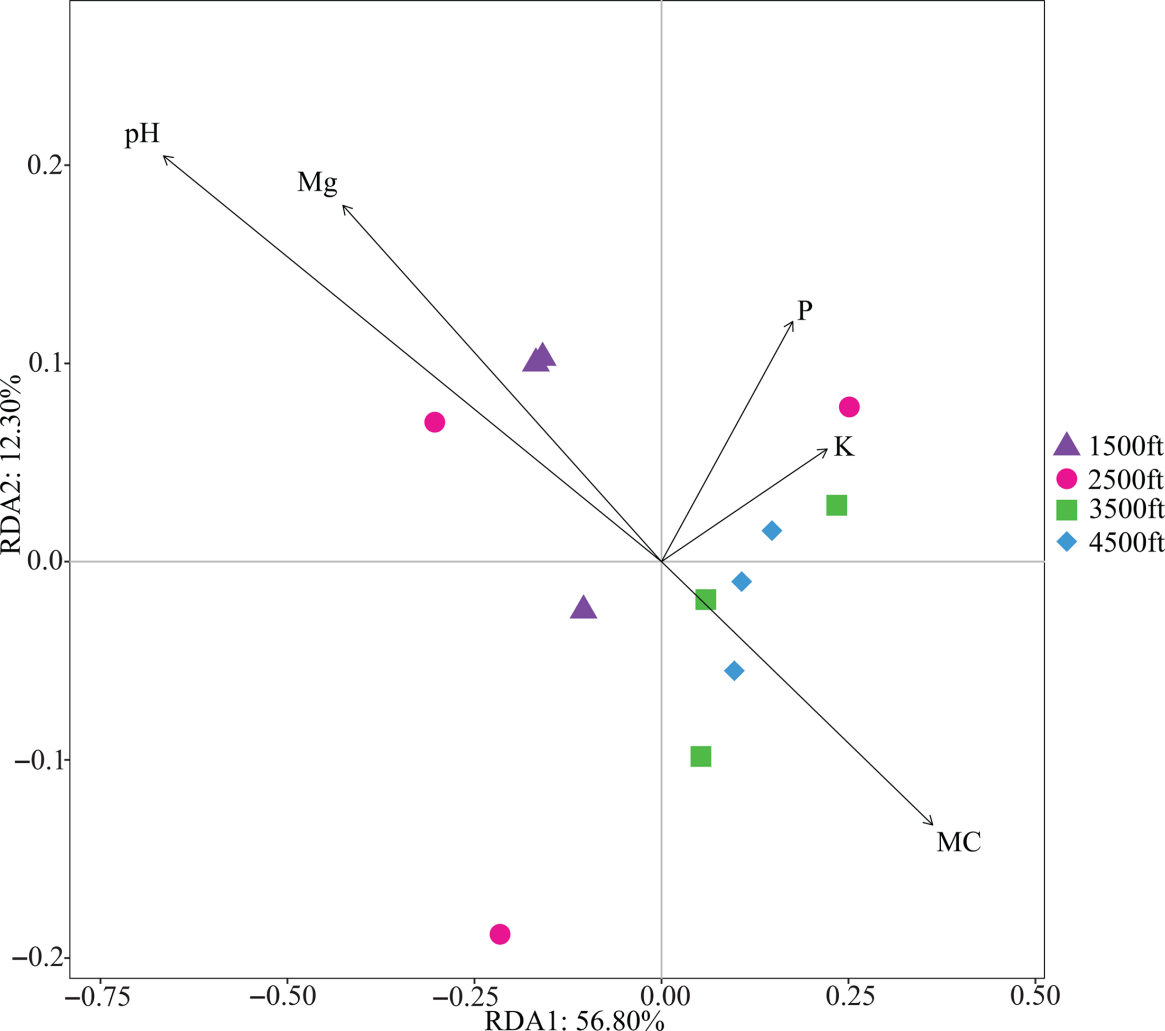
RDA of geochemical factors and bacterial communities in soil samples based on Euclidean distance computed at genus level grouped by altitude. Each point represents an individual replicate plot of soil sample at different altitudes. Each arrow indicates the impact of soil properties, and the vector length represents the degree of partial correlation of each soil factor with the RDA axes. Soil property abbreviations: K, potassium; MC, moisture content; Mg, magnesium; P, phosphorus.

The soil physicochemical properties of the sampling sites at four different altitudes in the NNF are shown in Table 1. Among these environmental factors, the magnesium content, calcium content, pH value, and cation exchange capacity all exhibited a decline as altitude increased. Redundancy analysis was used to assess the influence of different geochemical factors on the soil bacteria community structure at various altitudes, revealing that the 56.80% and 12.30% variabilities were explained by the primary and secondary correspondence for bacterial community composition (Fig. 5). The points representing bacteria at 1,500 ft formed a distinct cluster that was separated from those at 3,500 and 4,500 ft on the first principal component, suggesting that samples from low altitude differ from those of higher altitudes. This was partially consistent with the alpha diversity analysis (Shannon and Simpson indices), which showed that the diversity of 4,500-ft bacterial communities was significantly different from the 1,500-ft samples. Additionally, the points representing bacterial community structure of samples collected at 3,500 and 4,500 ft were closely clustered. Environmental factors demonstrated multivariate relationships with the diversity of bacterial communities in soil samples, and according to RDA followed by Mantel test, soil pH was the factor most correlated with bacterial community composition. Also showing significant positive correlation (*P* < 0.05) with general community structure (Table 2) were Mg content and cation exchange capacity, and these two soil properties had a strong positive correlation with each other (Table S9). The relative abundance of *Listeria* spp. in metagenomes detected by BLASTN was positively correlated with soil pH and cation exchange capacity (Table 2).

**TABLE 1:** Summary of soil physicochemical properties at four different altitudes in the Nantahala National Forest^*a*^

Altitude (ft)	Organic matter (%)	Total soil phosphorus (ppm)	Exchangeable cations			pH	Cation exchange capacity (meq/100 g)	Total organic carbon (%)	Gravimetric moisture content (%)
			K (mg/kg)	Mg (mg/kg)	Ca (mg/kg)				
1,500	19.0 ± 8.8	18.0 ± 7.6	82.7 ± 26.0	272.7 ± 111.5	2,163.3 ± 808.8	6.3 ± 0.5	14.0 ± 4.5	12.5 ± 6.3	27.6 ± 14.0
2,500	6.2 ± 2.3	8.0 ± 2.5	57.0 ± 4.4	79.3 ± 21.9	738.0 ± 521.7	5.6 ± 0.9	5.9 ± 2.1	3.6 ± 1.6	23.6 ± 5.3
3,500	57.6 ± 14.5	11.3 ± 3.5	104.7 ± 10.7	60.7 ± 3.2	217.0 ± 31.6	4.4 ± 0.1	4.9 ± 0.2	35.4 ± 9.4	167.3 ± 48.2
4,500	44.9 ± 16.9	31.7 ± 9.0	91.3 ± 19.6	41.7 ± 4.7	158.0 ± 24.3	4.2 ± 0.2	4.2 ± 0.2	28.0 ± 9.3	96.2 ± 16.7

^*a*^ Values presented were the average of triplicates (mean ± standard error, *n* = 3).

**TABLE 2:** The correlations of bacterial community composition analyzed by MG-RAST and *Listeria* abundance detected by BLASTN with custom database to different soil factors revealed by Mantel test^*a*^

	Bacterial community composition		*Listeria* abundance	
Soil property	Mantel statistic, *r*	Significance	Mantel statistic, *r*	Significance
Organic matter (%)	0.085	0.179	0.039	0.284
Total soil phosphorus (ppm)	−0.226	0.962	−0.105	0.771
K (mg/kg)	−0.039	0.562	−0.038	0.526
Mg (mg/kg)	0.290	**0.038**	0.267	0.066
Ca (mg/kg)	0.271	0.057	0.252	0.051
pH	0.535	**0.001**	0.428	**0.008**
Cation exchange capacity (meq/100 g)	0.368	**0.022**	0.334	**0.027**
Total organic carbon (%)	0.088	0.207	0.055	0.261
Moisture content (%)	−0.013	0.497	0.074	0.258

^*a*^ Significance values in bold indicate significance at *P* < 0.05.

## DISCUSSION

*Listeria* strains are free-living microbes that can be found in natural, agricultural, built-environmental soil, and food processing environments (59). In this work, we examined the prevalence of *Listeria* spp. in the NNF at four different altitudes, resulting in the isolation of 14 distinct *Listeria* strains from five species. Culture-independent metagenomics provided insight on the bacterial diversity in soil from the NNF and detected the *Listeria* spp. identified through the culture-dependent WGS method. For both methods, the occurrence of *Listeria* spp. was higher in soils from lower-altitude levels. Additionally, the soil pH and its cation exchange capacity were found to correlate with the presence of *Listeria* in the soil samples collected from the NNF. Through molecular characterization of *Listeria* isolates and analysis of soil metagenomes collected from a localized region of the NNF with significant elevation variation, this cross-sectional study provides a snapshot of *Listeria* diversity and distribution in the natural environment of the southern Appalachian region.

### Soil samples yielded several *Listeria* spp., including a recently described novel species

The most prevalent *Listeria* sp. recovered from the NNF was Lcs-ca, with 22 total isolates and eight distinct strains. For the well-known pathogenic species Lmo, only six isolates were obtained, consisting of three distinct strains. The three other species identified, Lma, Lsw, and Lbo, each had only a single distinct strain. Most strains (*n* = 12) were isolated from lower altitudes, including the Lma strain (2,500 ft), the Lsw strain (1,500 ft), all the Lmo strains (1,500 ft), and all but one of the Lcs-ca strains (five at 1,500 and two at 2,500 ft). In contrast, only a few strains (*n* = 3) were obtained from higher altitudes, including Lbo (3,500 ft), Lsw (4,500 ft), and a single Lcs-ca (4,500 ft). Interestingly, the single distinct Lsw strain was repeatedly isolated from two different samples representing two separate locations.

In their analysis of soil samples across the contiguous United States, Liao et al. (15) defined 12 *Listeria* genetic phylogroups with varying ranges of distribution. The strains isolated in the current study relate to four of those phylogroups: L2 (*L. monocytogenes* lineage II), L4 (*L. marthii*), L5 (*L. cossartiae*), and L12 (*L. booriae*). In that study, phylogroups L2 and L12 had broad distribution ranges that both included NNF, with L12 positive samples from the NNF region, further supporting the prevalence of *L. monocytogenes* lineage II and *L. booriae* in this region. In contrast, Liao et al. found that phylogroups L4 and L5 had very narrow distribution ranges, with L4 only being isolated in Kentucky, Pennsylvania, and New York, and L5 only in Alabama.

Additionally, the genus can be divided into two groups: *sensu stricto* and *sensu lato*. Most of the distinct strains (13 of 14) isolated in this study are in the *sensu stricto* clade, except for the single *L. booriae* isolate. The *Listeria sensu stricto* clade currently contains the pathogenic species *L. monocytogenes* and *Listeria ivanovii*, along with eight other species (12, 15, 60). Further investigation into the co-presence of *L. monocytogenes* and other non-pathogenic *sensu stricto* clade species in natural environments is required. These non-pathogenic species could potentially serve as index strains, whose presence reliably indicates the potential presence of related pathogenic species. Identifying such *Listeria* index strains could indicate the presence of *L. monocytogenes*. In food processing environments, the detection of *sensu stricto Listeria* sp. implies the potential presence of *L. monocytogenes* (11, 12).

*L. monocytogenes* was only isolated from a single site at a single altitude in the NNF. In our earlier investigation of a specific area within the Great Smoky Mountains National Park, which represents a subrange of the southern Appalachian Mountains, Lmo (specifically lineage II) emerged as the predominant *Listeria* spp. isolated from soil samples obtained at around 1,500 ft in elevation (11). In the current study, all three distinct Lmo strains also belonged to *L. monocytogenes* lineage II, and they were isolated at an altitude of 1,500 ft, underscoring the presence of this lineage in the southern Appalachian Mountains. These two different investigations were conducted in June (11) and September (this study). Sampling season may be a factor that influences the occurrence and diversity of *Listeria* spp. in the soil from these natural environments (8). However, it is important to note that this observation is based on only two cross-sectional studies, and more comprehensive, longitudinal research is needed to definitively establish the impact of seasonal variations on *Listeria* populations in these ecosystems. *Listeria* isolation is higher in spring due to higher humidity and remnants of decaying vegetation, but *Listeria* was detected during all seasons in a study investigating *Listeria* in soil and water samples collected in distinct geological and ecological sites in Austria (61). Although the isolation of Lmo lineage II strains in the southern Appalachian Mountain region may indicate their increased ability to survive in mountain conditions, no significant association has been identified between specific Lmo lineages and natural environments (62). Among the other 24 species which were detected by metagenomics without actual isolation, *Listeria welshimeri* and *Listeria innocua* were proposed to be present in this region, along with *L. monocytogenes* and *L. booriae*. However, no isolates of these species have been identified in the Southeastern United States previously.

The absence of other Lmo lineages, along with the clustering isolates from 1,500 ft (Fig. 2), could potentially be attributed to the inherent bias during culture-based selective enrichment procedures (63) or the subjective nature of selecting colonies on MOX and LMPM agar plates (11). Additionally, the number of samples may also explain the absence of other Lmo lineages. The BLEB used in this study is also used in the standard protocol for the enrichment of *Listeria* in the U.S. Food and Drug Administration *Bacteriological Analytical Manual*. There are also two other standard isolation protocols for *L. monocytogenes* strains, including the enrichment method in the USDA *Microbiology Laboratory Guidebook* and the International Organization for Standardization method (64, 65). Although no standard enrichment methods are completely free of bias (66), BLEB was shown to allow more *Listeria* spp. to grow to detectable levels than other media in standard protocols (64). Additionally, the limited number of isolates selected during isolation-based methods could impact the interpretation of *Listeria* diversity in environmental samples. To address this limitation, a combination of culture-dependent and culture-independent techniques, such as metagenomic sequencing, would enhance the accuracy and depth of *Listeria* diversity assessments in nature.

*L. swaminathanii* is a novel *sensu stricto* species that was originally and subsequently isolated from the Great Smoky Mountains National Park (11, 12). That, along with the isolation of this species in the present study, provides support that *L. swaminathanii* may be prevalent in the southern Appalachian Mountain region (13). In the NNF, Lsw had relatively broader habitat breadths along altitude levels. Previous research has shown that almost identical *Listeria* isolates can be obtained from geographically unrelated environments (8). While all the other identical *Listeria* strains were obtained from the same soil samples, the *L. swaminathanii* strain was isolated from soil collected from two different sampling locations. The re-isolation of this strain at different geographical locations at the same time indicates it may be widespread in the NNF (67). It was proposed that natural environments may allow for more efficient bacterial dispersal, leading to a higher possibility of isolating identical strains at different sampling locations. Furthermore, the capability of *Listeria* to establish resilient populations in diverse conditions is essential for its wide distribution in natural environments (7).

*L. cossartiae* was isolated from both the lowest and highest elevations, indicating its potential widespread presence in the region. Only a single strain of each of *L. marthii* and *L. booriae* was isolated in this study. Despite their low prevalence, the detection of these species in both the current study area and the Great Smoky Mountains National Park suggests their regional presence (11). Although these species appear to be less prevalent, their detection emphasizes the need for more comprehensive genomic data. Currently, there is an uneven representation of *Listeria* genomes in public databases such as NCBI. In comparison to the extensive collection of over 53,200 genomes of Lmo available on NCBI, the number of *L. cossartiae* genomes is currently limited to 13, while *L. marthii* has only 38 genomes. Our study contributes to the available isolates and genomes of *L. marthii* and *L. cossartiae*, thereby offering valuable resources for expanding the understanding of phylogroups in future research on *Listeria* distribution.

### *Listeria* prevalence and soil microbiome composition changed with altitude

Soil is a competitive environment for bacteria, making it essential to understand the background microbiome in soil environments where *Listeria* is present (68). In this study, Proteobacteria and Actinobacteria were the most abundant phyla in soil samples at all different altitudes. Several previous studies that examined the soil bacterial community composition in the Nantahala Mountain range of the southern Appalachian Mountains of North Carolina showed that Proteobacteria and Acidobacteria were the most abundant bacterial phyla, while Actinobacteria was also present in soil metagenomes with considerable abundance (>1% of all sequences) (69–71). A dominance of Proteobacteria and Actinobacteria could be associated with low pH of soil environment (Table 1) (72). In the current study, the composition of soil bacterial communities at the class level shifted with altitude (Fig. 3B). The shift of bacterial community composition with elevation has also been noted in other mountain regions such as Mount Shergyla at southeastern Tibetan Plateau in China and Mount Fanjing in southern China and is attributed to changes in soil physicochemical properties (73, 74). In future studies, conducting ecological network analysis of *Listeria* strains, along with background bacterial community in soil, might provide a comprehensive understanding of which taxa are associated with *Listeria* spp. and how they interact in that environment. This approach could assist in the identification of concomitant microorganisms that serve as indicators for the presence of *Listeria* spp. or *L. monocytogenes* in the natural environment.

In a comprehensive study investigating bacterial diversity on a global scale, it was observed that the diversity of soil bacterial community was negatively correlated with altitude (75). However, in a specific mountain area such as Mount Fuji in Japan, Mount Da-an in central Taiwan, and Mount Taibai in eastern mainland China, the bacterial diversity may exhibit a non-linear or hump-backed trend in relation to altitude (76–78). In the NNF, the bacterial diversity exhibited a significant variation across different altitudinal gradients. The composition and diversity of bacteria result from intricate interactions involving multiple geographical and meteorological factors (78, 79). Although higher soil microbial diversity can be disadvantageous for the survival of foodborne pathogens in soil due to the competition of available nutrients (80, 81), *Listeria* spp. were still predominantly isolated from lower-altitude soil with relatively high bacterial diversity in this study, indicating that altitude might be an important factor for *Listeria* isolation from soil in the NNF. Elevation was also observed as highly associated with *Listeria* isolation from natural environments in New York state (20). Solar radiation is stronger at higher altitudes due to the thinner atmosphere, leading to the reduced growth and survival of microorganisms (10). Another study found that soil samples collected from the eastern Alps at lower altitudes (0–500 m) had a much higher occurrence of *Listeria* (25.27%) compared to higher altitudes (3.85% at 500–1,000 m and 0.86% at >1,500 m) (61).

### Metagenomic sequencing can be used to identify *Listeria* spp. in natural environments

Metagenomic methods provide benefits over traditional culture-based methods that can facilitate faster identification and characterization of foodborne pathogens in different kinds of environmental and clinical samples for source tracking and outbreak prevention. This type of analysis holds great promise in the surveillance of *Listeria monocytogenes* and other foodborne pathogens, and it has been used for foodborne pathogen detection in manufacturing environments to track shifts in pathogen distribution during food production (82). Genomic data of *Listeria* also provide strong support for the application of metagenomics in detecting pathogen hazards in both foods and food processing environments (83). In this study, the metagenomic reads assigned to the *Listeria* genus were further identified at the species level by BLASTing against a custom *Listeria* genome database, and they were congruent with the five different *Listeria* spp. identified by culture-based WGS methods. These results indicated that metagenomic analysis of the soil bacterial community was effective in detecting the *Listeria* spp. that had been isolated and identified through the culture-dependent method. However, the soil samples contained a greater diversity of *Listeria* spp. than was determined through the culture-dependent method, indicating that either the read classification methods may have misclassified some reads at the species level or some were unable to be classified. Basic methodologies, such as laboratory protocols for total DNA isolation from soil samples, may also affect the outcomes of the metagenomic analysis (84, 85), though no significant variation in OTU richness was observed between the two different soil sample sizes used with the DNeasy PowerSoil Pro Kit and the DNeasy PowerMax Soil Kit (86).

For the detection of enteric pathogens in clinical samples for surveillance and foodborne outbreak investigation, metagenomic results have demonstrated consistency with those from culture-dependent WGS methods (30). Metagenomics has also seen limited use for source tracking of foodborne pathogen outbreaks, offering a proof of concept for linking isolates from human cases to the strain from the implicated food source (87). Metagenomic tools are sometimes able to distinguish non-pathogenic bacteria from pathogens in clinical samples with high specificity and sensitivity compared to conventional testing methods (88). However, the co-existence of pathogenic and non-pathogenic *Listeria* spp. in natural environments complicates the task of tracking *Listeria* pathogens precisely, making effective surveillance of *Listeria* challenging (82).

In this study, only a small proportion of metagenomic reads (<0.2%) from soil samples were assigned to *Listeria*. The reliability of the metagenomic results was improved by the identification of *Listeria* through genomic sequencing of isolates recovered from the soil samples. The coverage and accuracy of the database of *Listeria* genomes are critical for achieving a comparable level of resolution to that of isolate-based WGS methods. Through sequencing genomes of newly isolated *Listeria* spp. from diverse environments, the representation and diversity of *Listeria* genomes available in publicly accessible databases can be improved. In turn, this can increase the efficacy of *Listeria* surveillance through metagenomic strategies, as reads can more reliably be mapped to a reference genome.

Additionally, the success of metagenomic testing is highly dependent on the depth of sequencing (88). While *Listeria* is commonly found in soils, it is a low-abundance subpopulation compared to the overall soil bacterial community composition (10, 15). In future studies, sequencing metagenomes at greater depths can be used for *de novo* assembly, enabling the generation of contigs that provide a more accurate representation of the *Listeria* isolates recovered from environmental samples. Metagenomic techniques usually provide molecular evidence of pathogens. However, obtaining *Listeria* isolates from metagenomic-positive samples could be technically challenging due to the difficulty in recovering viable but non-culturable or dead cells (89). Reconstruction of metagenomic-assembled genomes through ultra-deep sequencing from natural samples may overcome the limitation of culture-dependent methods in detecting unculturable *Listeria* (89). It may also provide functional information on unculturable *Listeria*, thus enhancing our understanding of their survival mechanisms and transmission. Additionally, the detection of virulence factors from specific pathogenic bacteria can also be used as an indicator that the environment is a reservoir for clinically relevant bacteria (90).

### Soil pH and cation exchange capacity were significantly correlated with *Listeria* abundance in soil metagenomes

Soil serves as a natural reservoir for a variety of bacteria, including *Listeria* spp., and soil physicochemical properties serve as the primary drivers of soil bacterial communities (71). In this study, soil pH, Mg content, and CEC were correlated with bacterial community composition in soil collected from the NNF (Table 2). The pH of soil is a major driver of the structure of soil microbial communities (91), with acidic soil being less advantageous for microbial growth than soil with a pH close to neutral (10). Other studies investigating bacterial composition in the NNF of western North Carolina found a significant correlation between bacterial community composition and soil pH (69, 71), consistent with results obtained in the current study conducted in the same region. The soil factors affecting soil bacterial community structure are site-specific, and different types of ecosystems usually support unique microbial communities (92). Similar to our findings, Mg content was identified as a significant factor influencing soil bacterial community composition in other international forest regions, including tropical forests in Kenya and Mountain Ash forests of southeastern Australia (93, 94), while cation exchange capacity was also found to be significantly correlated with soil bacterial community diversity in other international forest locations such as forests in the Karst region of subtropical Southwest China, forests in Southern Europe, and subtropical forests in southeastern China (95–97).

The persistence of *Listeria* in natural environments plays a major role in the transmission of pathogenic *Listeria* strains to food processing environments (98). Identifying factors that contribute to the prevalence of *Listeria* in soil environments is crucial for understanding the environmental selection and risk of transmission to human and animal hosts. The pH of soil is the most significant factor influencing abundance of *Listeria* reads in soil from the NNF (Table 2). In a study evaluating the survival of *L. monocytogenes* and *L. innocua* in surface soil from a forest in Ireland, the authors hypothesized that low soil pH may contribute to shorter survival times (68). It was shown that *L. monocytogenes* could not persist for more than 6 days in forest soil in France with low pH (99). In the Appalachian Mountains, soil at high elevations has low buffering capacity to acidification by atmospheric acid deposition (72). The acidic pH of soil from higher altitudes (Table 1) may explain the lower abundance of *Listeria* spp. in those soils in the current study. Besides unfavorable natural conditions, lower human and animal population densities have also been proposed as explanations for lower abundance of *Listeria* at higher altitudes (61). Moreover, CEC has been recognized as one of the abiotic factors associated with the presence and fate of *L. monocytogenes* in soil environments (100, 101). In this study, CEC was also shown to be correlated with the abundance of *Listeria* in soil, possibly due to its strong influence on the overall composition of the soil bacterial community. Another significant factor affecting soil bacterial community composition was Mg content in the soil of the NNF. Although no correlation was observed between the Mg content and abundance of *Listeria* in the soil, Mg content was significantly correlated with CEC of soil (Table S9). This is expected, as Mg^2+^ is one of the exchangeable cations in soil (102).

The correlation statistics in this study are challenging because of the small sample size, which is due in part to the large spatial range of the natural environment and the limited spatial resolution of soil properties inherent in the observation methodology (103). It is important to recognize the limitations imposed by the small sample size on the Pearson correlation analysis. With a limited number of data points, the correlation coefficients become more vulnerable to the influence of outliers or extreme values (104). Additionally, the small sample size increases risk of Type II errors (false negatives) and leads to wider confidence intervals for the correlation coefficients, making it challenging to accurately estimate the strength of these relationships (105, 106). The stability of the correlations is also compromised, as small changes in the data set could result in substantial fluctuations in the observed correlations (107). Given these limitations, we advise caution in over-interpreting the magnitude of the observed correlations, presenting these results as preliminary indications of potential relationships. We emphasize the need for validation through larger-scale studies. To mitigate these issues, we employed conservative statistical approaches, such as Bonferroni correction, and presented appropriate caveats of our findings. We strongly recommend that future studies build upon our work with larger sample sizes to validate and extend our preliminary observations, enabling more robust and generalizable conclusions about the relationships and patterns we have identified.

Different geographical factors have been identified as associated with *Listeria* occurrence in farm environments, including soil moisture (highly correlated with *Listeria* isolation) (20). Higher soil moisture increases the survival of *L. monocytogenes* and other foodborne pathogens (108). In another study analyzing the nationwide prevalence of *Listeria* spp. in the United States, higher soil moisture and organic matter content were correlated with the prevalence of *Listeria* in soil samples (15). However, in the current study, soil moisture was not associated with *Listeria* detection in this particular natural environment. The geographical factors associated with *Listeria* prevalence are still only partially understood due to the limited sampling from natural environments. Further characterization of the genomes of the *Listeria* isolated from natural environments may provide functional insight into their capability to persist in those environments. Machine learning model protocols such as random forest modeling can be used to predict the factors affecting the prevalence of *Listeria* spp. in a specific environment. This information can aid in the development of control strategies to reduce *Listeria monocytogenes* transmission from natural environments to farms and food products (6).

### Conclusions

In this study, we investigated the presence of *Listeria* in the soil of the NNF and evaluated the relationship between *Listeria* abundance, diversity of bacterial communities, and soil geochemical properties. The soil samples yielded 42 *Listeria* isolates, which were sequenced by culture-dependent WGS and determined to consist of 14 distinct strains and 5 *Listeria* spp. These newly isolated strains have expanded knowledge on the diversity of the genus *Listeria* in natural environments. Culture-independent metagenomics was performed to elucidate the ecology of *Listeria* in natural soil environment, revealing the prevalence and diversity of *Listeria* in soil from the NNF. The genomes of different *Listeria* spp. were also used for downstream analysis of metagenomics, enhancing the resolution of molecular detection of *Listeria* strains in the soil from the NNF. Both culture-dependent and culture-independent analyses indicated a higher occurrence of *Listeria* spp. in soil samples from relatively lower altitudes. Soil pH and cation exchange capacity were found to correlate with both the bacterial community composition and *Listeria* distribution in soil of the NNF. This study highlights the potential to use soil metagenomes to provide information on the occurrence of *Listeria* spp. in natural environments, thereby contributing to future applications of metagenomics in foodborne pathogen surveillance and facilitating the rapid identification and accurate prediction of transmission risks.

## Data Availability

Genomic data of *Listeria* isolates are available at the National Center for Biotechnology Information (NCBI) under BioProject PRJNA942302, including raw sequencing reads from the Sequence Read Archive and assemblies from GenBank. Metagenomic sequencing reads were submitted to NCBI under BioProject PRJNA942317.

## SUPPLEMENTAL MATERIAL

Supplemental figures spectrum.01712-24-s0001.docx Fig. S1 and S2.

Supplemental tables spectrum.01712-24-s0002.xlsx Tables S1 to S9.

ASM does not own the copyrights to Supplemental Material that may be linked to, or accessed through, an article. The authors have granted ASM a non-exclusive, world-wide license to publish the Supplemental Material files. Please contact the corresponding author directly for reuse.
