# Supplementary material for: Whole-genome sequencing and metagenomics reveal diversity and prevalence of Listeria spp. from soil in the Nantahala National Forest
Source: Microbiol Spectr. 2024 Dec 9;13(1):e01712-24. doi: 10.1128/spectrum.01712-24 (PMC11705966; doi:10.1128/spectrum.01712-24)
Supplement: Supplemental figures — Fig. S1 and S2. [file spectrum.01712-24-s0001.docx]

**Supplementary Material**

**Whole-Genome Sequencing and Metagenomics Reveal Diversity and Prevalence of *Listeria* spp. from Soil in the Nantahala National Forest**

Jia Wang^a^, Claire N. Schamp^a^, Lauren K. Hudson^a^, Harleen K. Chaggar^a^, Daniel W. Bryan^a^, Katie N. Garman^b^, Mark Radosevich^c^, Thomas G. Denes^a,^*

^a^Department of Food Science, The University of Tennessee, Knoxville, Tennessee 37996, USA

^b^Tennessee Department of Health, Nashville, Tennessee 37243, USA

^c^Department of Biosystems Engineering and Soil Science, The University of Tennessee, Knoxville, Tennessee 37996, USA

Jia Wang and Claire N. Schamp contributed equally to this work. Author order was determined in order of decreasing seniority.

*Corresponding Author

Email address: tdenes@utk.edu


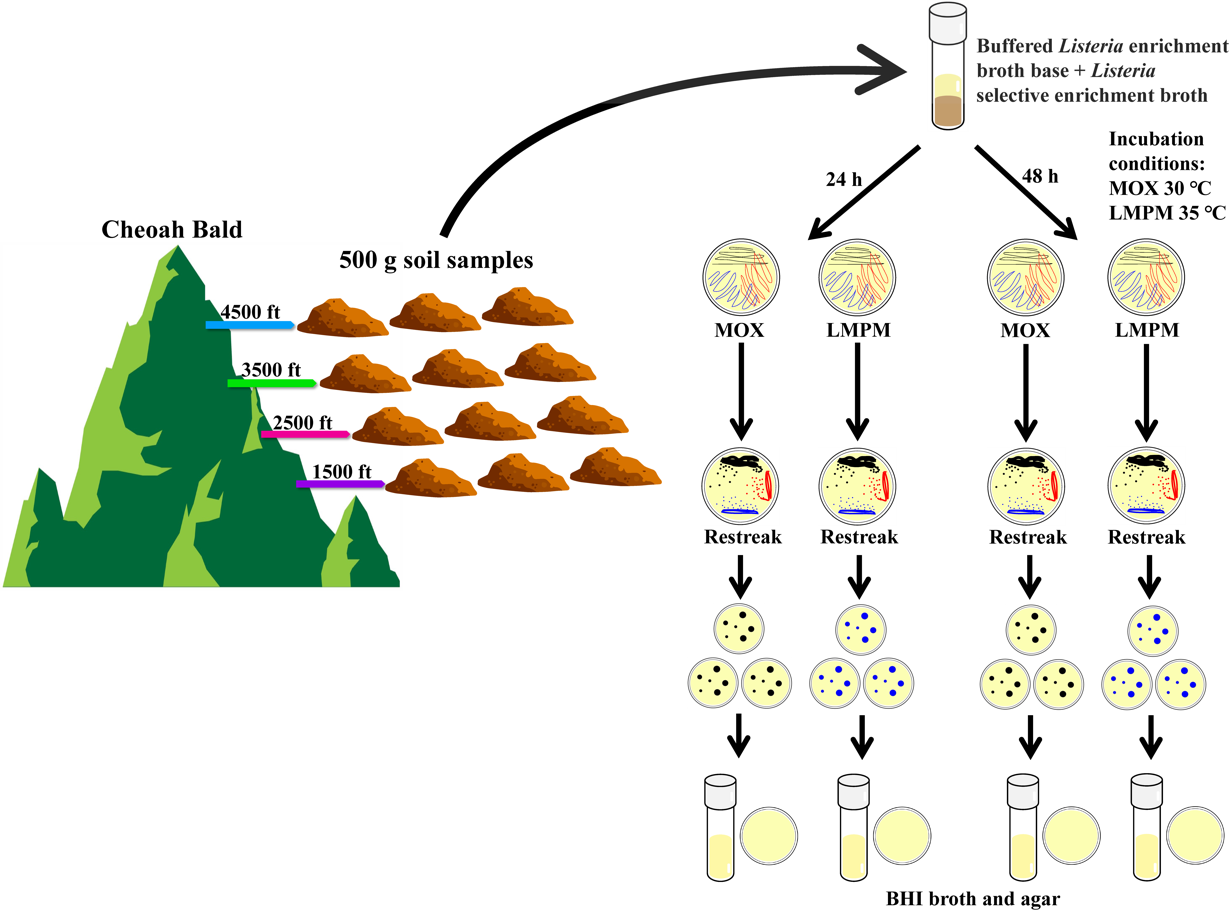


**Figure S1.** Schematic representation of soil sample collection at four different altitudes in the Nantahala National Forest and the isolation of *Listeria* spp. strains from soil samples.


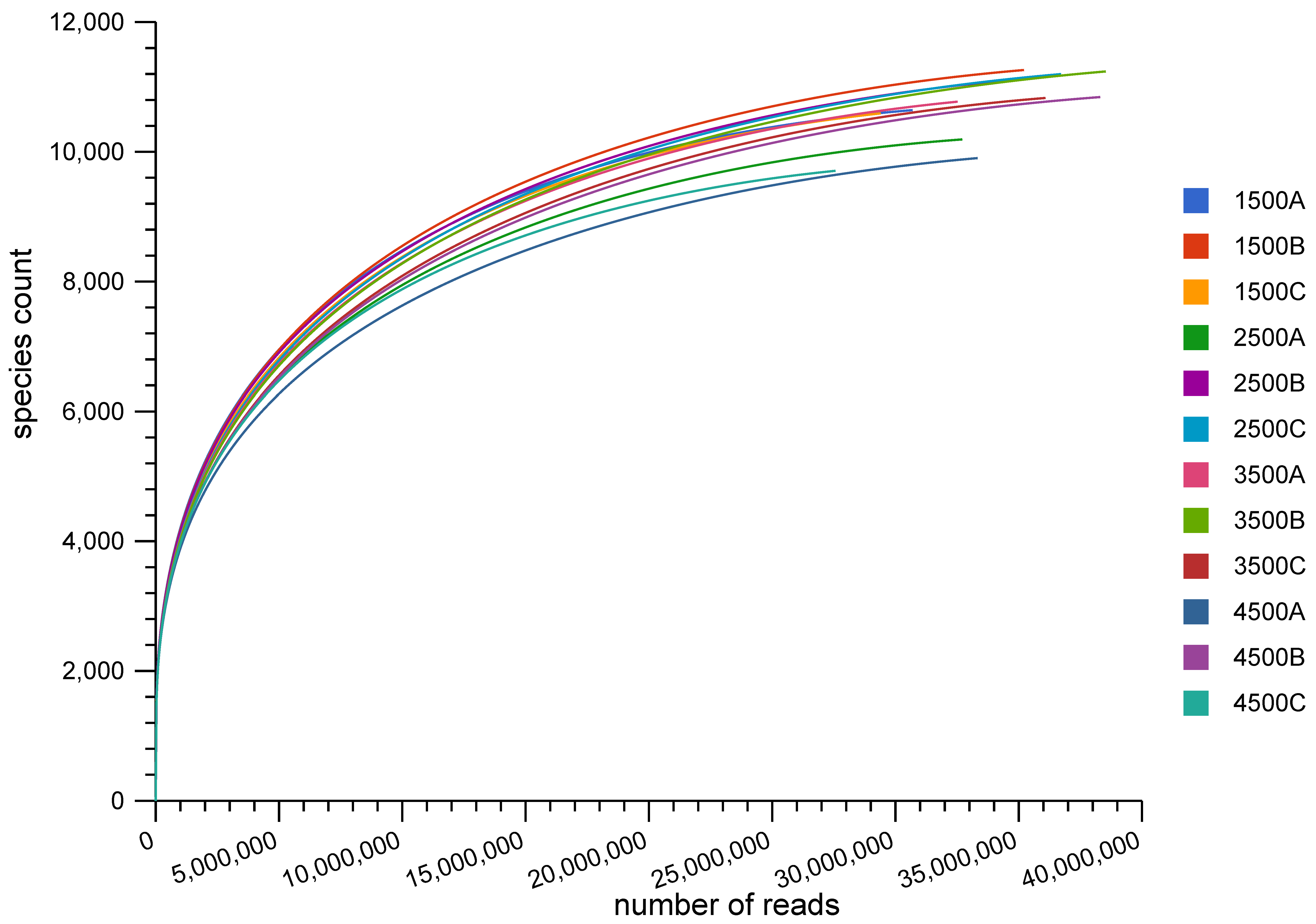


**Figure S2.** Rarefaction curves for metagenome sequences of bacterial communities in soil samples from the Nantahala National Forest at different altitudes. The alphanumeric labels represent the altitude and replicate of each sample, respectively. The sampling site elevations are indicated by the number: 1,500 ft (1500), 2,500 ft (2500), 3,500 ft (3500), 4,500 ft (4500). The letter following each elevation number represents parallel samples collected at the same elevation.
